# Accelerometer Cut Points for Physical Activity Assessment of Older Adults with Parkinson’s Disease

**DOI:** 10.1371/journal.pone.0135899

**Published:** 2015-09-02

**Authors:** Håkan Nero, Martin Benka Wallén, Erika Franzén, Agneta Ståhle, Maria Hagströmer

**Affiliations:** 1 Department of Neurobiology, Care Sciences and Society, Division of Physiotherapy, Karolinska Institutet, Stockholm, Sweden; 2 Department of Physical Therapy, Karolinska University Hospital, Stockholm, Sweden; University of Pennsylvania Perelman School of Medicine, UNITED STATES

## Abstract

**Objective:**

To define accelerometer cut points for different walking speeds in older adults with mild to moderate Parkinson’s disease.

**Method:**

A volunteer sample of 30 older adults (mean age 73; SD 5.4 years) with mild to moderate Parkinson’s disease walked at self-defined brisk, normal, and slow speeds for three minutes in a circular indoor hallway, each wearing an accelerometer around the waist. Walking speed was calculated and used as a reference measure. Through ROC analysis, accelerometer cut points for different levels of walking speed in counts per 15 seconds were generated, and a leave-one-out cross-validation was performed followed by a quadratic weighted Cohen’s Kappa, to test the level of agreement between true and cut point–predicted walking speeds.

**Results:**

Optimal cut points for walking speeds ≤ 1.0 m/s were ≤ 328 and ≤ 470 counts/15 sec; for speeds > 1.3 m/s, they were ≥ 730 and ≥ 851 counts/15 sec for the vertical axis and vector magnitude, respectively. Sensitivity and specificity were 61%–100% for the developed cut points. The quadratic weighted Kappa showed substantial agreement: κ = 0.79 (95% CI 0.70–0.89) and κ = 0.69 (95% CI 0.56–0.82) for the vertical axis and the vector magnitude, respectively.

**Conclusions:**

This study provides accelerometer cut points based on walking speed for physical-activity measurement in older adults with Parkinson’s disease for evaluation of interventions and for investigating links between physical activity and health.

## Introduction

The positive health effects of physical activity for the general population are countless [1], and physical activity and exercise can be especially advantageous for individuals with Parkinson’s disease (PD); effects include general improvement of health, prevention of depression, a decrease in fatigue, improved functional performance and drug efficiency, and an optimization of the dopaminergic system [2]. PD is the second-most common age-related neurodegenerative disease, after Alzheimer’s disease [3]. The cardinal symptoms of PD are tremor, bradykinesia, rigidity, and postural instability [4]. Together with non-motor symptoms such as depression and sleep disturbance, these symptoms might influence physical activity and sedentary behaviour [5–7].

One of the components of physical activity important for desirable health outcomes is intensity. The effects of increased physical activity (e.g., structured exercise) seen in individuals with PD, such as improved neuroplasticity and greater step length, increase in proportion to the intensity level [8]. Hence, intensity is of great interest when measuring physical activity in a PD population. When evaluating physical activity interventions, population-specific methods of measurement must be chosen. Compared to subjective measurements (e.g., questionnaires or diaries), objective measures (e.g., accelerometry) do not require recall. Recall can constitute a great cognitive task [9], and the most common activity performed by older adults is light-to-moderate-intensity physical activity—which is the most difficult to recall [9, 10]. Therefore, objective measurements such as accelerometry are preferable when measuring the activity of older adults [11].

Accelerometry also has the potential to provide information about the intensity, duration, and frequency of physical activity [12]. However, to explore intensity, the accelerometer output (i.e., counts) needs to be translated to more physiologically meaningful information [13] or to another appropriate prognostic measure. This can be achieved via validation against a measure of energy expenditure or via calibration against standardized activities, such as walking or running [14], often yielding accelerometer cut points that reflect the activity’s intensity [15]. Although most studies have included an energy-expenditure measurement (such as indirect calorimetry) as a criterion measure, accelerometers assess a biomechanical aspect of physical activity. Therefore, researchers have suggested interpreting intensity in the biomechanical domain—for instance, by referring derived cut points to walking speed [16].

Altered gait patterns and walking variability are commonly observed in older adults [17, 18], and since the most commonly reported physical activity of older adults is walking [19], this has been an essential part of existing calibration studies in this population [20–22]. Although similarities exist, older adults are not representative of individuals with PD. Cardinal features of PD include gait dysfunction, as in shortened stride length, increased variability, freezing, and shuffling of gait [23]. Naturally, this will alter accelerometer output. Hence, existing cut points will not be applicable to the PD population, and to our knowledge none have previously been produced specifically for individuals with PD. In addition, since walking speed can reflect various physiological processes and has the potential to predict future health and mortality [24], it may serve as a calibration measure. Therefore, the objective of this study was to define and cross-validate accelerometer cut points for different walking speeds in older adults with mild to moderate PD.

## Method

### Participants

A sample of 31 older adults (13 women) with PD living in Stockholm, Sweden, was recruited through advertisements via patient organizations and at local clinics. Sample size was based on previous calibration studies in healthy adults [13]. Participants were contacted by telephone, informed of the study, and briefly screened by interview for inclusion and exclusion criteria. The inclusion criteria were an age of ≥ 60 years, a clinical diagnosis of idiopathic PD (Queens Square Brain Bank criteria) [25], and mild to moderate disease severity (Hoehn & Yahr score II and III) [26]. Participants were excluded if they were in need of walking assistance or aids or if they were unable to complete a total of nine minutes of independent indoor walking. Calibration (development of accelerometer cut points) was performed on the total sample, and to investigate the validity of the resultant cut points, a leave-one-out cross-validation was performed.

### Ethics statement

All participants provided written informed consent. The study was approved by the Regional Board of Ethics in Stockholm (DNR 2006/151-31, 2009/819-32, and 2011/37-32).

### Procedure

The study was conducted at the Karolinska Institutet in Stockholm, Sweden. All parts of the protocol were led by an experienced physiotherapist, and each participant was tested on a single occasion. At arrival, written informed consent was provided and signed before height and weight were measured using a stadiometer and scale. Subsequently, each participant was interviewed, and for descriptive purposes information regarding his or her activities of daily living was assessed with the Unified Parkinson’s Disease Rating Scale part II (UPDRS-ADL) [27], which is a 13-item questionnaire graded on a 4-point scale that is summarized into a total score (0–52, where a higher score equals more disturbance). Freezing of gait (FOG) was assessed with the Freezing of Gait Questionnaire (FOGQ), specifically developed for individuals with PD [28, 29]. Thereafter, the UPDRS part III (motor part) was performed to score disease and symptom severity and to define Hoehn and Yahr disease stage [30]. The daily levodopa equivalent dose (LED) was calculated as described by Tomlinson et al. [31]. Body mass index (BMI) was calculated using kg/m^2^; information about age and years since diagnosis was collected through the interviewing process. Lastly, information regarding comorbidities and PD-related symptoms was collected.

Next, participants briefly practiced walking at participant-defined brisk, normal, and slow speeds and estimating perceived exertion using the Borg’s Rating of Perceived Exertion (RPE) scale [32] so as to become familiar with the instructions. Participants were fitted with an accelerometer (sampling rate set at 30 Hz) attached to an elasticized belt and worn around the waist, the unit placed over the lateral side of the hip. The accelerometer used was a triaxial Actigraph GT3X+ (Actigraph, Pensacola, FL), which is widely used for the objective measurement of physical activity [13]. It is a lightweight (19 grams) accelerometer that records time-varying changes in acceleration in three planes (vertical, anteroposterior, and mediolateral) and specifies these accelerations in an arbitrary unit (counts) in either axis or as a composite vector magnitude (VM). The GT3X+ has been validated against total energy expenditure and tested for reliability [33]. Accelerometer specifications of greater detail have been published elsewhere [34]. Participants were also fitted with a Polar FT4 (Polar Electro, Finland) heart-rate monitor belt around the chest.

To attain different walking speeds the participants were instructed to walk a total of three times at the self-defined speeds of brisk, normal, and slow (in that order). This order was selected since pilot testing had revealed that fatigue might influence brisk walking speed. Walking was performed in a circular indoor hallway measuring 210 metres, the distance measured and marked beforehand using a measuring wheel (Trade Quip TQ, Melbourne, Australia) and taped floor markings. Participants walked for three minutes each bout, and then rested until they were confirmed recovered and until their heart rate had lowered to their pre-walking level. Rather than incorporating a shorter bout duration, three minutes of walking was selected to attain a steadier and more stable walking pace, hence increasing the measurements’ accuracy [35]. Before each walking bout and after three minutes of walking, participants estimated exertion on the RPE scale, and heart rate was documented to monitor and ensure participant recovery. To record actual walking start time, an external timepiece was used, synchronized with the accelerometer’s clock. After each bout, walking distance was documented.

Through measured walking distance and walking duration, walking speed was calculated in km/h and m/s. Accelerometer data was downloaded using the software ActiLife 6 (Actigraph, Pensacola, FL, USA) and summarized over 15 seconds. The ActiLife default filter setting was used, according to previously published recommendations [36].

### Data analysis

**Fig 1:**
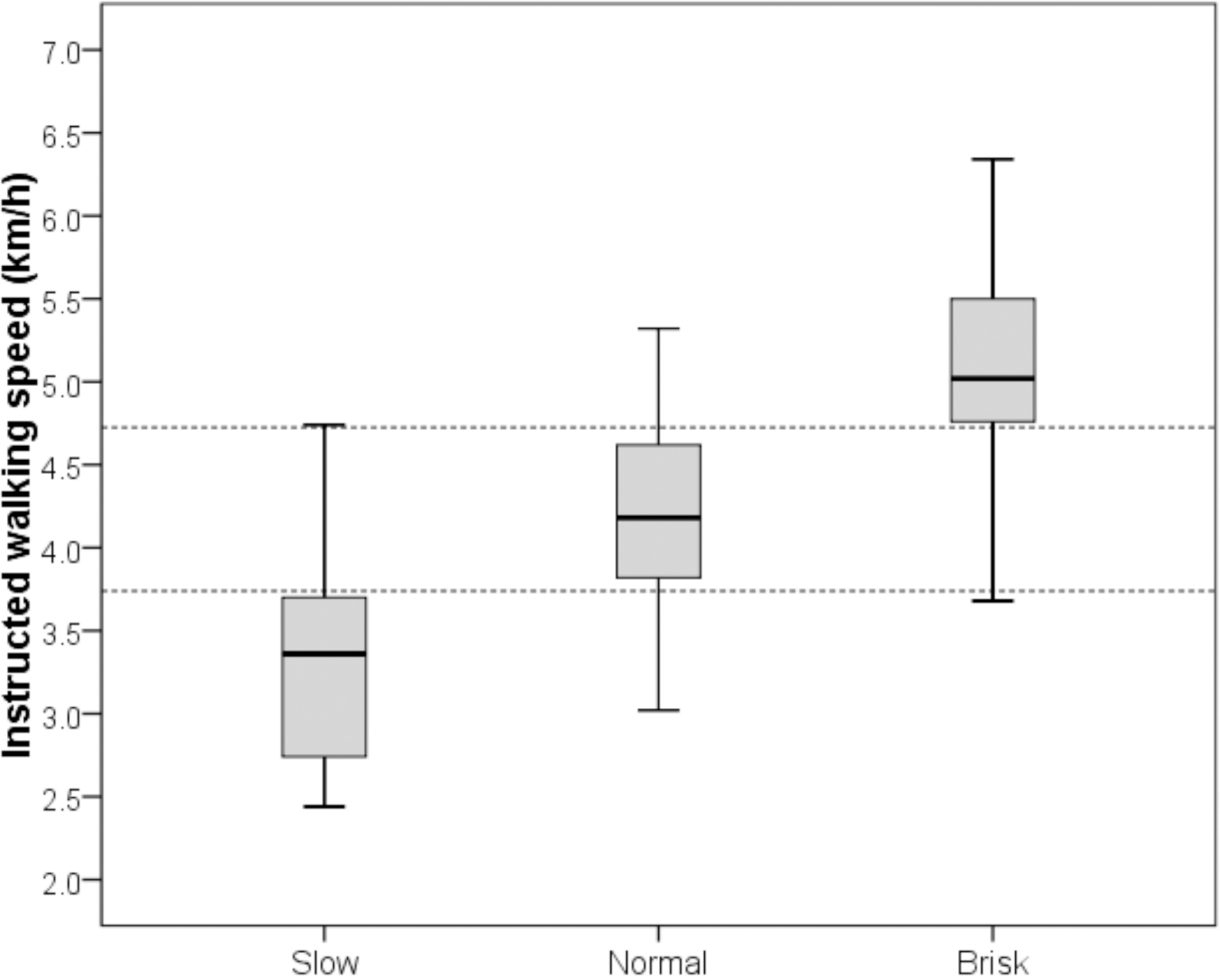
Percentiles of participant-defined speeds used for restructuring. The upper dotted line represents the 25th percentile of brisk walking, and the lower dotted line marks the 75th percentile of slow walking.

For the development of accelerometer cut points for different walking speeds in the y-axis (vertical) and vector magnitude (VM) counts, data were restructured using the 75th percentile of participant-defined slow walking (3.74 km/h or 1.04 m/s) and the 25th percentile of participant-defined brisk walking (4.73 km/h or 1.31 m/s) as the low- and high-speed thresholds, respectively (Fig 1).

Receiver operating characteristic (ROC) curves were used and coordinates of the curve visually inspected, to find cut points with optimal sensitivity and specificity, here presented in percentages (%) [37]. In order to minimize the risk of slowly walking individuals being mis-categorized, false negatives were preferred over false positives for the brisk walking cut-point (i.e. highest possible specificity). For the same reason, maximal sensitivity, which increases the probability for true positives, was prioritized for the slow walking cut-point [38]. This type of prioritization is common for accelerometer calibration studies [39, 40]. Area under the curve (AUC) with confidence intervals, are also presented. When defining accelerometer cut points, indices calculated from a ROC curve provide empirical basis for determining the most appropriate threshold value or cut point [41]. AUC values of ≥ 0.90 are considered excellent, values between 0.80–0.89 are deemed good, values 0.70–0.79 are fair, and those < 0.70 are considered poor [42]. A leave-one-out cross-validation was then conducted on n-1 participants, followed by determination of agreement between speed classified by derived cut points and actual walking speed of the left-out participant. This was repeated through the total sample. Walking speeds were categorized according to the restructured speeds, and a quadratic weighted Cohen’s Kappa was calculated [43].

Descriptive statistics for participants and main outcomes are presented as the mean (SD) for continuous variables, the median, and the 25th–75th percentiles for non-normally distributed or ordinal data. Data was analysed using SPSS version 22 for Windows (SPSS Inc., Chicago, IL, USA). The level of significance was set at p ≤ 0.05.

## Results

One individual was excluded because of sudden illness during testing. Hence, the sample totaled 30 participants (13 women). One individual performed three minutes of brisk walking and one minute of normal walking but thereafter did not continue owing to fatigue. This individual is included in the analysis but with corresponding missing data. Descriptive data are presented in Table 1.

**Table 1:** Participant characteristics.

Descriptive variables	Total (N = 30)
Age (years)	73 ± 5.4
BMI (kg/m^2^)^a^	24.6 ± 3.3
PD duration (years)	6 (3–9) †
UPDRS-ADL	17 (13–19)
H & Y score 2/3	15 (50) / 15 (50)
LED^1^	693 (378–832) †
UPDRS motor	36 ± 8
FOGQ^2^	5 (2–9)

Characteristics of the participants presenting mean ± SD, median (25th–75th percentile) if data are non-normally distributed or ordinal, or number of cases (%).

^a^BMI = Body mass index. H & Y score = Hoehn and Yahr score. LED = daily Levodopa equivalent dose. FOGQ = Freezing of Gait Questionnaire.

† = Non-normally distributed.

Descriptive data of walking speed intervals are presented in Table 2. Mean ± SD heart rate per walking speed intervals was 89 ± 17, 95 ±18 and 106 ± 19, respectively. The medians (25th–75th percentile) for rated exertion on the RPE scale per walking speed (≤ 1.0 m/s, 1.1–1.30 m/s, and > 1.3 m/s) were 13 (9–13), 13 (11–13) and 13 (13–15).

**Table 2:** Counts and walking speed per speed intervals.

Outcome variables	≤1.04 m/s	1.05–1.30 m/s	≥1.31 m/s
	n = 32	n = 28	n = 28
Y-axis counts / 15 sec^a^	256 ± 150	574 ± 144	819 ± 217
VM counts / 15 sec^b^	427 ± 230	679± 176	911 ± 232
Walking speed (km/h)	3.2 ± 0.4	4.2± 0.3	5.3± 0.5
Walking speed (m/s)	0.89 ± 0.11	1.17 ± 0.08	1.47 ± 0.14

Mean counts / 15 sec ± SD in y-axis and vector magnitude (VM), and mean walking speed per speed interval.

^a^Vertical axis counts per 15 seconds.

^b^Vector magnitude counts per 15 seconds.

Table 3 shows generated cut points for the lowest and highest walking speeds based on the ROC analysis, along with their respective AUC values. Consequently, for the walking speed 1.05–1.30 m/s, estimated counts/15 sec were 329–729, and 471–850 for the y-axis and VM, respectively.

**Table 3:** Generated cut points per walking speed intervals.

Axis	Speed^a^	Sensitivity (%)	Specificity (%)	AUC (95% CI)^b^	Cut point^c^
Y	≤ 1.04	100	75	0.940 (0.882–0.889)	≤ 328
	1.05–1.30				
	≥ 1.31	68	82	0.826 (0.716–0.936)	≥ 730
VM	≤ 1.04	89	66	0.816 (0.708–0.923)	≤ 470
	1.05–1.30				
	≥ 1.31	64	82	0.784 (0.663–0.905)	≥ 851

Sensitivity, specificity, AUC, and cut points (per 15 s) in the y-axis (vertical) and vector magnitude (VM) for the defined walking speeds.

^a^Walking speed in m/s.

^b^Area under the curve; CI = confidence interval.

^c^Counts/15 sec.

The leave-one-out cross-validation showed 74% and 64% absolute agreement between actual walking speed and speed defined by the derived cut points for the y-axis and VM, respectively. The quadratic weighted Kappa showed substantial agreement: κ = 0.79 (95% CI 0.70–0.89) for the y-axis, and κ = 0.69 (95% CI 0.56–0.82) for the VM [44].

## Discussion

To our knowledge, this is the first study to provide accelerometer cut points for different walking speeds in older adults with PD. Optimal cut points for walking speed ≤ 1.0 m/s were < 328 and < 470 counts/15 sec, and for walking speed > 1.3 m/s they were > 730 and > 851 counts/15 sec for the y-axis and VM, respectively. The leave-one-out cross-validation showed substantial agreement between true and cut-point-defined walking speeds. The resultant cut points may be used to quantify physical activity behaviour in this population and to ascertain regular free-living walking speed for older adults with mild to moderate PD.

Accelerometer cut points for different types of units and populations can be found in the literature [39, 45, 46], including cut points for the Actigraph GT3X and GT3X+ for older adults [47, 48]. Results from the GT3X-specific study report VM cut points at 2,751 counts per minute for moderate-intensity (3–5.99 metabolic equivalents; METs) and 9,359 counts per minute for vigorous-intensity physical activity (6–8.99 METs) [47]. Compared to the present study’s results (for 1.1–1.3 m/s and > 1.3 m/s), cut points from the aforementioned study are slightly higher. This could owe to calibration-method differences (different epoch setting, treadmill vs. hallway walking) as well as different population-sample characteristics; for instance, a PD-altered gait involving a shorter step length would increase step frequency [49] in comparisons to a healthy older adult walking at the same speed. Likewise, it is important to consider that in the aforementioned study intensity was assessed using indirect calorimetry (using a stationary device), an analysis which is not included in the method of the present study.

Calibration utilizing indirect calorimetry is a common procedure in many studies for ascertaining energy expenditure (and defining intensity) for a specific work load or task [15]. But the accuracy of portable indirect calorimetric measures (which is essential if participants walk freely and not on a treadmill) for older adults can be questioned, since overestimation of energy expenditure during rest has been reported (an average 30% overestimation), where bias increases with increasing expenditure. The method might also lead to discomfort for the older participant [50]. Therefore, this method was not employed in the present study. Furthermore, measured heart rate and participant’s perceived exertion proved to be unreliable measures demonstrated by great variability in our sample, and therefore not appropriate for intensity determination. Reasons for this could be that PD affect the autonomic nervous system which regulates heart rate, and that many participants were treated with drugs affecting heart rate and/or did not understand how to rate their exertion. Consequently, exact energy expenditure of walking during the different speeds is unknown, and a comparison to similar populations is of interest. It has been shown that walking at a speed of ≥ 1.31 m/s generates near equal numbers of counts per minute in individuals with multiple sclerosis (MS) [46] and in adults 20–60 years old [51], as in the present study. Moreover, this walking speed requires a metabolic cost of 5.9 METs in older adults without PD, signifying close to vigorous activity [12]. In addition, leisurely hallway walking (unknown walking speed) of older adults (measured METs of 4.1) generates roughly the same number of counts per minute as the present study’s walking speed of 1.1–1.3 m/s does. Despite these similarities, cut points generated via indirect calorimetry for individuals with MS are different from those in the present study. This once again highlights the possible effects of altered gait in PD. Hence, the ability to approximate intensity via walking speed in this group is uncertain and needs to be studied further.

In line with the present study’s results, previous studies have shown that older adults with PD walk at a speed of 1.1 m/s and 1.5 m/s when walking at normal and maximum walking speed, respectively [52]. Evidence also supports that this group has lower normal and maximum walking speeds than age-matched healthy older adults do [53]. The lowered walking speed of older adults with PD is of clinical interest since walking speed is considered to be a consistent risk factor for disability, cognitive impairment, falls, and mortality in community-dwelling older adults. According to contemporary evidence rendered by large-scale studies, a usual walking speed of < 1.0 m/s (3.6 km/h) is a cutoff value that identifies individuals at high risk of negative health-related outcomes, death, and hospitalization within one year [54, 55]. Further, findings have shown that together with previous falls and FOG during the last month, a walking speed of < 1.1 m/s (< 3.96 km/h) predicts future falls in people with PD [56]. Since these cutoff values are comparable to the lower walking speed in the present study, derived cut points may reflect not only the physiological intensity of walking to a certain degree but also a possible risk of adverse events for certain individuals in this population.

This study has some important strengths worth consideration. The calibration includes both regular and brisk walking, which is important for calibration purposes and, as previously noted, might reflect risk of adverse events. Moreover, walking in a corridor instead of on a treadmill, which has been a commonly used method of calibration, might better reflect an individual’s natural walking pattern, especially among older adults with less experience of treadmill walking and with a chronic disease that affects gait.

Generating cut points in a 15-second epoch (summarizing data over 15 seconds) instead of applying the commonly used 1-minute epoch increases the possibility of capturing shorter bursts of activity or movement that a 1-minute epoch setting might miss [16]. This seems appropriate within the PD population, which exhibits common symptoms such as tremor, FOG, and dyskinesia. In addition, the availability of cut points in both the y-axis and VM might be considered a strength. However, since the vertical axis cut-points have higher sensitivity and specificity, we recommend these in favor of the VM based thresholds, even though previous studies have shown VM to more accurately classify physical activity intensity [47].

Although other options are available, generating cut points for physical activity quantification was the method of choice. Alternatives such as pattern recognition take advantage of more features of the raw accelerometer data and might reduce under- and overestimation of energy expenditure [57], thus increasing accuracy [58]. Still, this method is not commonly used. Though it is in some respects a more limited method, utilizing cut points is an easy and accessible method when analysing monitored free-living physical activity [33]. Until more-advanced options become available and are standardized for the research community and clinicians alike, cut points might be the most user-friendly and most readily available option.

This study’s cross-validation on derived cut points may also be considered a strength. The quadratic weighted Cohen’s Kappa that was used assigns less weight to agreement if categories are further apart [44]; hence, it takes into account whether the cut-point-defined speed is close to the actual speed or far from it. Showing substantial agreement, the cross-validation indicates the accuracy of generated cut points for activity quantification in the population of older adults with PD.

### Study limitations

The recruitment of participants was based on potential candidates who answered advertisements, showed interest in participation, and fit the criteria. Combined with an inclusion criterion of mild to moderate disease severity, this might pose a threat to external validity. The reason for this criterion is that in this stage of disease, the individuals are still ambulatory. And since these results will be used for analysis on a larger balance-training intervention study in older adults with PD [59], the inclusion criteria were matched. Also, as walking speed may differ in different cultural and geographical environments, it is suggested that these results be verified in other cultures or countries. Despite these limitations, the sample characteristics (age, gender distribution, disease severity) match reported numbers of prevalence and incidence in Europe and northern Sweden fairly well [60, 61].

Another possible limitation is that the derived cut points are specific to the Actigraph GT3X+, which hinders the use of these with other models and brands of accelerometers. Since variation in calibration results might occur between different models of Actigraph [15], it is recommended that the resultant cut points only be used for the GT3X+. The Actigraph is a commonly used unit for physical-activity measurement [34], and when calibrating an accelerometer and utilizing the type-specific and arbitrary unit of counts, unfortunately this limitation is unavoidable. Furthermore, even when using uniform equipment and standardized procedure for data collection, varying methods of analysis can alter results. This needs to be taken into consideration when using accelerometers for physical activity measurement.

## Conclusion

This study provides Actigraph GT3X+ cut points defining different walking speeds in older adults with PD. The resultant cut points may be used to evaluate interventions and to investigate links between physical activity and health, as well as to collect data of walking speed during daily living in this population.
